# Housing, Social Support, and Transition in Connecticut’s Money Follows the Person Program

**DOI:** 10.1093/geroni/igaf122.3004

**Published:** 2025-12-31

**Authors:** Amal Alsamawi, Asmita Aasaavari, Christine Bailey, Kathy Kellett, Tyfany Fabian, Alexandra Sanders, Julie Robison, Ellis Dillon

**Affiliations:** University of Connecticut, Farmington, Connecticut, United States; University of Connecticut, Farmington, Connecticut, United States; University of Connecticut, Farmington, Connecticut, United States; University of Connecticut, Farmington, Connecticut, United States; University of Connecticut, Farmington, Connecticut, United States; University of Connecticut, Farmington, Connecticut, United States; University of Connecticut, Farmington, Connecticut, United States; University of Connecticut, Farmington, Connecticut, United States

## Abstract

Money Follows the Person (MFP) is a Medicaid program that helps older adults move from Long-Term Care institutions to the community. In this study, we examined the role of housing and social support on older adults’ ability to move out of institutional settings through the experiences of MFP stakeholders in Connecticut. Preliminary findings from semi-structured interviews with older adults who are eligible or applied to MFP (n = 25), caregivers (n = 16) and MFP professional staff (n = 43) are presented. Qualitative analysis included key word searching, inductive coding, and thematic analysis. Older adults and their caregivers reported that access to housing increased opportunities to leave nursing facilities. However, access to housing was not always sufficient. An older adult with dementia who was reinstitutionalized recounts losing her apartment because she did not have familial support, “I lost everything... My niece never went to the apartment”. Housing related assistance from family members included moving in with a family member, support finding a place, and facilitating the move, “My family is the one that went out and found this apartment and they moved all my stuff”. An MFP staff described the challenge for older adults, in particular people with dementia to transition if they do not have family support – “It’s possible but it is really hard…going home with family is definitely the easier route”. The presence of informal support shapes transition and can improve access to housing opportunities. Innovative approaches to housing are imperative for the growing population of older adults with minimal family support.

